# LtpA, a CdnL-type CarD regulator, is important for the enzootic cycle of the Lyme disease pathogen

**DOI:** 10.1038/s41426-018-0122-1

**Published:** 2018-07-09

**Authors:** Tong Chen, Xuwu Xiang, Haijun Xu, Xuechao Zhang, Bibi Zhou, Youyun Yang, Yongliang Lou, X. Frank Yang

**Affiliations:** 1 Key Laboratory of Laboratory Medicine, Ministry of Education of China, School of Laboratory Medicine, Wenzhou Medical University, 325000 Wenzhou, Zhejiang China; 2 Department of Microbiology and Immunology, Indiana University School of Medicine, Indianapolis, IN 46202 USA; 3 Department of Anesthesiology, The First Affiliated Hospital, College of Medicine, Zhejiang University, 310003 Hangzhou, China; 4 State Key Laboratory of Rice Biology and Ministry of Agriculture Key Laboratory of Agricultural Entomology, Institute of Insect Sciences, Zhejiang University, 310058 Hangzhou, China

## Abstract

Little is known about how *Borrelia burgdorferi*, the Lyme disease pathogen, adapts and survives in the tick vector. We previously identified a bacterial CarD N-terminal-like (CdnL) protein, LtpA (BB0355), in *B. burgdorferi* that is preferably expressed at lower temperatures, which is a surrogate condition mimicking the tick portion of the enzootic cycle of *B. burgdorferi*. CdnL-family proteins, an emerging class of bacterial RNAP-interacting transcription factors, are essential for the viability of *Mycobacterium tuberculosis* and *Myxococcus xanthus*. Previous attempts to inactivate *ltpA* in *B. burgdorferi* have not been successful. In this study, we report the construction of a *ltpA* mutant in the infectious strain of *B. burgdorferi*, strain B31-5A4NP1. Unlike CdnL in *M. tuberculosis* and *M. xanthus*, LtpA is dispensable for the viability of *B. burgdorferi*. However, the *ltpA* mutant exhibits a reduced growth rate and a cold-sensitive phenotype. We demonstrate that LtpA positively regulates 16S rRNA expression, which contributes to the growth defects in the *ltpA* mutant. The *ltpA* mutant remains capable of infecting mice, albeit with delayed infection. Additionally, the *ltpA* mutant produces markedly reduced spirochetal loads in ticks and was not able to infect mice via tick infection. Overall, LtpA represents a novel regulator in the CdnL family that has an important role in the enzootic cycle of *B. burgdorferi*.

## Introduction

Bacterial CarD N-terminal-like (CdnL) protein, a defining member of the CarD_CdnL_TRCF protein family, is part of an emerging class of bacterial RNAP-interacting transcription factors^1–5^. The CarD_CdnL_TRCF protein family contains two types of proteins, CarD-type and CdnL-type. CarD is a global transcriptional regulator that contains two domains: a C-terminal AT-hook DNA-binding domain resembling eukaryotic high-mobility group A (HMGA) proteins and an N-terminal transcription repair coupling factor (TRCF) domain that interacts with RNA polymerase. CdnL proteins are more common and widespread among bacterial species. They have a N-terminal TRCF domain but lack a C-terminal AT-hook DNA-binding domain^2^. Recent structural studies show that CdnL contains two sub-domains: CdnLNt, which consists of five β strands and interacts with RNAP, and CdnLCt, which consists of five α-helices and contains a solvent-exposed nonpolar-basic patch that is involved in DNA binding^4^^,6–8^.

Although the presence of CdnL-type CarD transcription factors is widespread among bacterial species^9^, our understanding of their function remains limited. In Mycobacteria, CdnL-type CarD was shown to regulate ribosomal RNA (rRNA) transcription in tuberculosis (Mtb)^5^, and its expression is upregulated during several stress conditions including oxidative stress, starvation, and antibiotic treatment^5,10^. CdnL-type CarD in Mycobacteria is essential for cell viability. *Myxococcus xanthus* contains both CarD and CdnL. In *M. xanthus* CdnL, but not CarD, is essential for cell viability^2^. *Bacillus* species appear to have two copies of CdnL proteins. The inactivation of one of the *cdnL* genes did not affect cell replication in *B. subtilis and B. cereus* but resulted in defects in the repair and outgrowth of heat-damaged spores in *B. cereus*^11^. Additionally, *cdnL* expression was upregulated in the vegetative cells of *B. cereus* in response to stress conditions^12^.

*Borrelia burgdorferi*, the Lyme disease pathogen, is maintained in nature via an enzootic cycle between ticks and mammals. Although much is known regarding how *B. burgdorferi* modulates its gene expression to adapt to mammalian host environments; the process of spirochetal adaptation in its tick host is poorly understood^13–15^. Two pathways have been identified that are critical in regulating spirochetal adaptation in ticks, the c-di-GMP signaling pathway^16–21^ and the (p)ppGpp stringent response pathway^22–25^. c-di-GMP is important for the ability of *B. burgdorferi* to utilize nutrients in ticks, including glycerol, chitobiose, and *N*-acetyl glucosamine^16,20,26,27^, and is involved in the chemotaxis, motility, cell envelope structure and osmolarity, all of which are important for the survival of *B. burgdorferi* in ticks^17–20,28,29^. (p)ppGpp is required for persistence in ticks and is involved in the expression of glycerol uptake and metabolism (*glp*) operon as well as that of other tick-associated genes such as *ospA*, *bicA*, and *pncA*^23,25^.

We previously reported that LtpA (BB0355) in *B. burgdorferi* is homologous to CdnL-type CarD. LtpA is most highly expressed at ambient temperature (23 ℃), which is a surrogate condition for the tick environment^30^. Previous attempts to generate an *ltpA* mutant in virulent *B. burgdorferi* strain 297 were unsuccessful, which has hampered the effort to elucidate the role of LtpA in the enzootic cycle of *B. burgdorferi*. Herein, we report the successful construction of an *ltpA* deletion mutant in the virulent *B. burgdorferi* strain B31 clone 5A4NP1. We demonstrate that LtpA is indispensable for spirochetal viability, but is required for the optimal survival of *B. burgdorferi* in the tick vector.

## Results

### LtpA is differentially expressed by temperature and growth phase

**Fig. 1:**
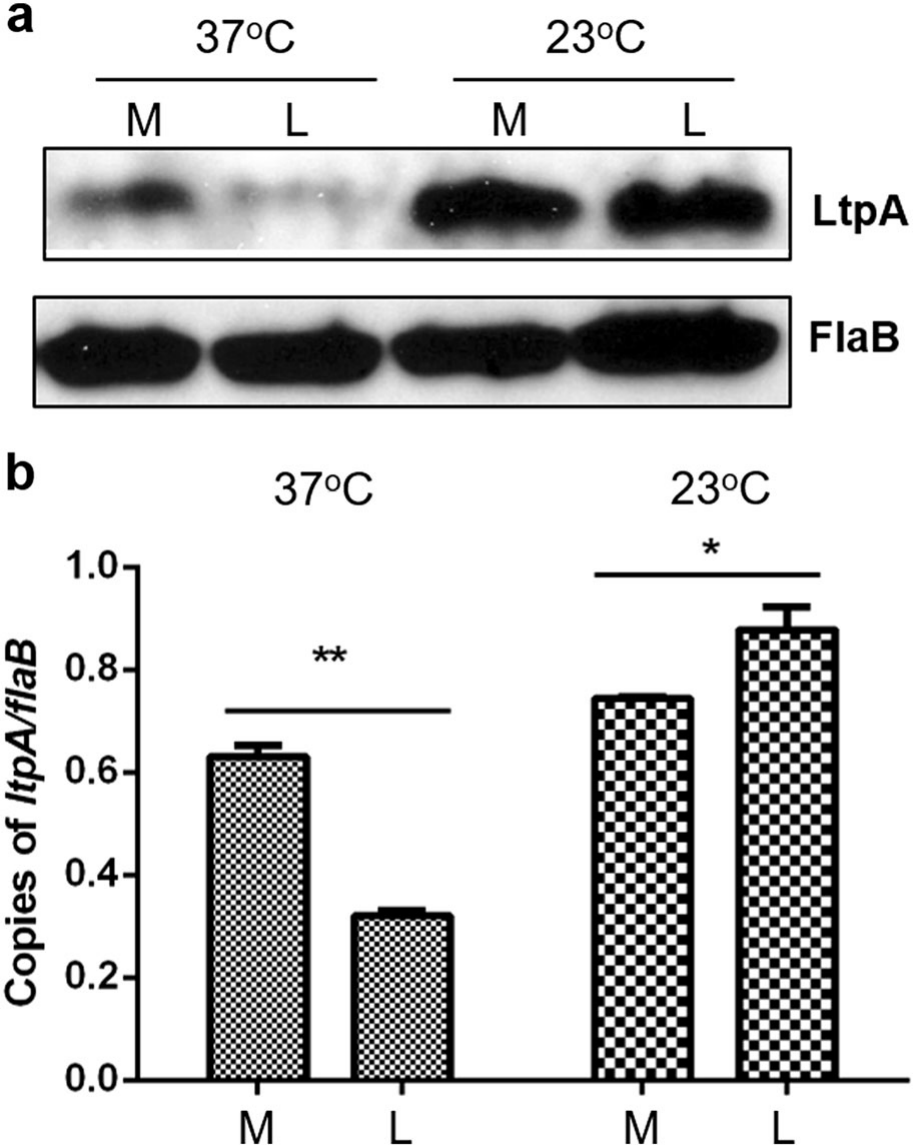
Influence on LtpA level by temperature and growth phase. *B. burgdorferi* strain 5A4NP1 was cultured in BSK-II medium at 37 °C or 23 °C. Spirochetes were collected at the mid-log phase (M, 10^7^ spirochetes/ml) or late-log phase (L, 10^8^ spirochetes/ml) then processed for immunoblot (**a**) and qPCR analyses (**b**). Levels of FlaB protein and *flaB* mRNA served as internal controls. *, p < 0.05; **, p < 0.01

To investigate the regulation of *ltpA*, we examined spirochetes cultivated at different temperatures (23 or 37 ℃) and harvested at different growth phases (mid-logarithmic (10^7^ cells/ml) or a late-logarithmic phase (10^8^ cells/ml)). As reported previously, little to no LtpA was detected in *B. burgdorferi* when cultivated under conditions mimicking mammalian infection (37 ℃, late-logarithmic phase), while significant LtpA was detected in spirochetes grown in conditions mimicking tick colonization (23 ℃) (Fig. 1a)^30^. When harvested at the mid-logarithmic phase, LtpA was readily detected even at 37 °C, suggesting that in addition to temperature, cell density also regulates *ltpA* expression at 37 °C. This finding was supported by qRT-PCR results (Fig. 1b). At 23 ℃, LtpA appeared to be abundantly produced, regardless of the growth phase (Fig. 1a, b).

### Construction of the *ltpA* mutant and the complementation strain

**Fig. 2:**
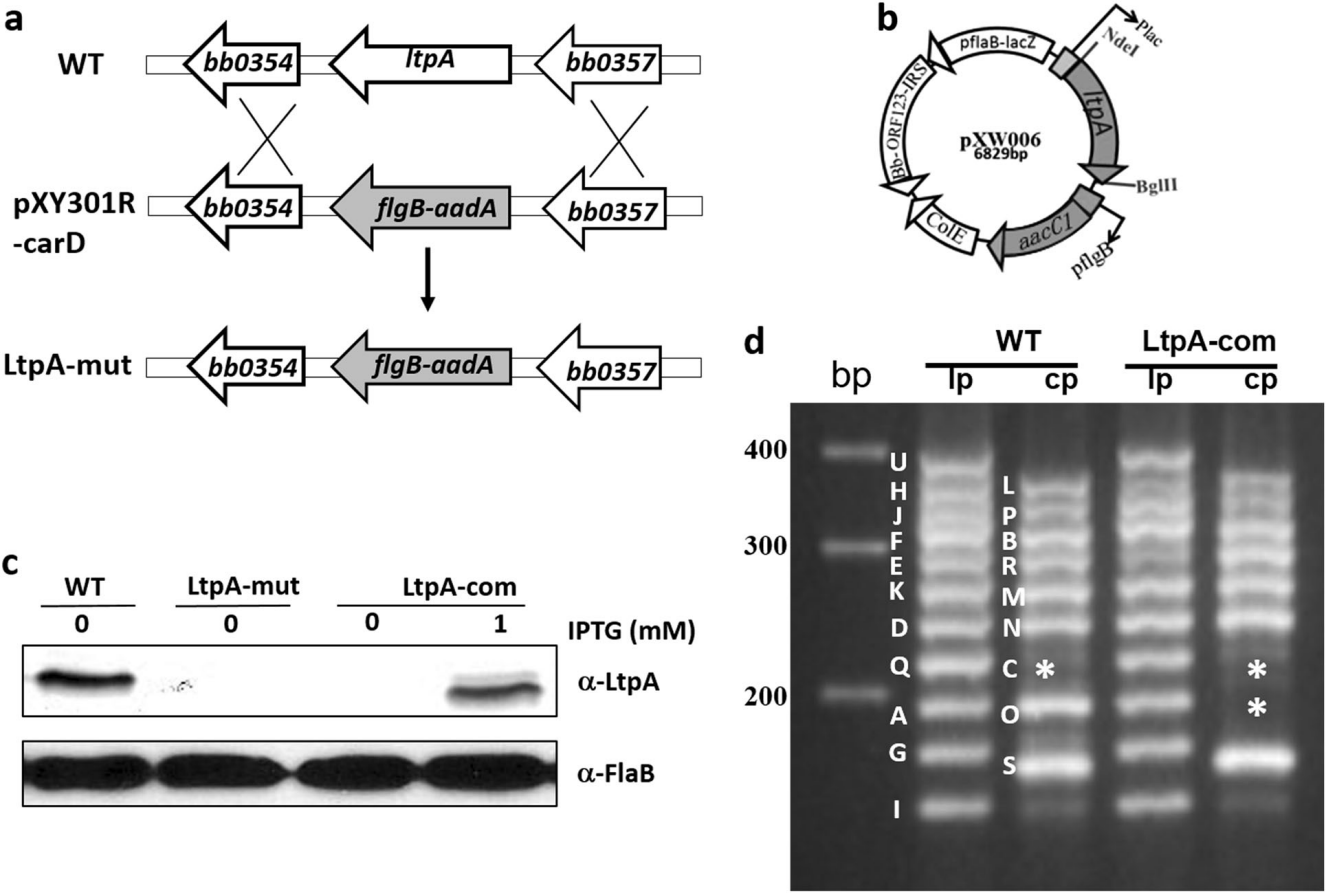
Construction of the *ltpA* mutant and the complementation strain. **a** Strategy for constructing the *ltpA* mutant. WT: genomic structure of *ltpA*. pXY301R-carD: the suicide vector used for inactivation of *ltpA*. LtpA-mut: the *ltpA* mutant. **b** Diagram of the shuttle vector used for complementation. **c** *B. burgdorferi* strains were cultured in BSK-II medium with or without 1 mM IPTG. Spirochetes were collected at the mid-log phase (10^7^ spirochetes/ml) then processed for immunoblotting analysis. WT wild-type *B. burgdorferi* strain 5A4NP1, Ltp-mut the *ltpA* mutant, LtpA-com the complementation strain. **d** Endogenous plasmid profiles of wild-type (WT) and complementation strains (LtpA-com). The *ltpA* mutant has an identical plasmid profile to that of the WT (not shown). * indicates the band corresponding to plasmid cp32-7 that is missing in LtpA-com

To investigate the function of LtpA, we constructed an *ltpA* mutant using allelic exchange in a low passage, infectious strain of *B. burgdorferi* strain B31 clone 5A4NP1. A suicide vector pXY301R-CarD was constructed with an *aadA* gene (which confers streptomycin-resistance) that was flanked by upstream and downstream regions of *ltpA* (BB0355, Fig. 2a). pXY301R-CarD was transformed into infectious *B. burgdorferi* strain B31 clone 5A4NP1, where it replaced the *ltpA* gene. Four streptomycin- and kanamycin-resistant clones were obtained; these clones were subjected to PCR analyses for confirmation of the correct *ltpA* deletion (data not shown). One clone that maintained identical endogenous plasmids to those of the parental strain 5A4NP1 was chosen for complementation. For complementation, a pBSV2-derived shuttle vector with a gentamycin-resistance cassette carrying a wild-type copy of *ltpA* driven by an IPTG-inducible promoter, pXW006 (Fig. 2b), was transformed into the *ltpA* mutant. Of note, complementation using a shuttle vector harboring a constitutively expressed *flaB* promoter-driven *ltpA* was not successful despite multiple attempts (data not shown), implying that the overexpression of *ltpA* may be deleterious to *B. burgdorferi*. The loss of LtpA in the *ltpA* mutant and the restoration of LtpA production in the complementation strain (when grown in the presence of 1 mM IPTG) were confirmed by immunoblotting (Fig. 2c). One complementation clone that had a virtually identical endogenous plasmid profile to that of the parental strain 5A4NP1, with the exception of losing one of the cp32 plasmids, cp32-7 (Fig. 2d), was selected for further study.

### The *ltpA* mutant has reduced growth in vitro

**Fig. 3:**
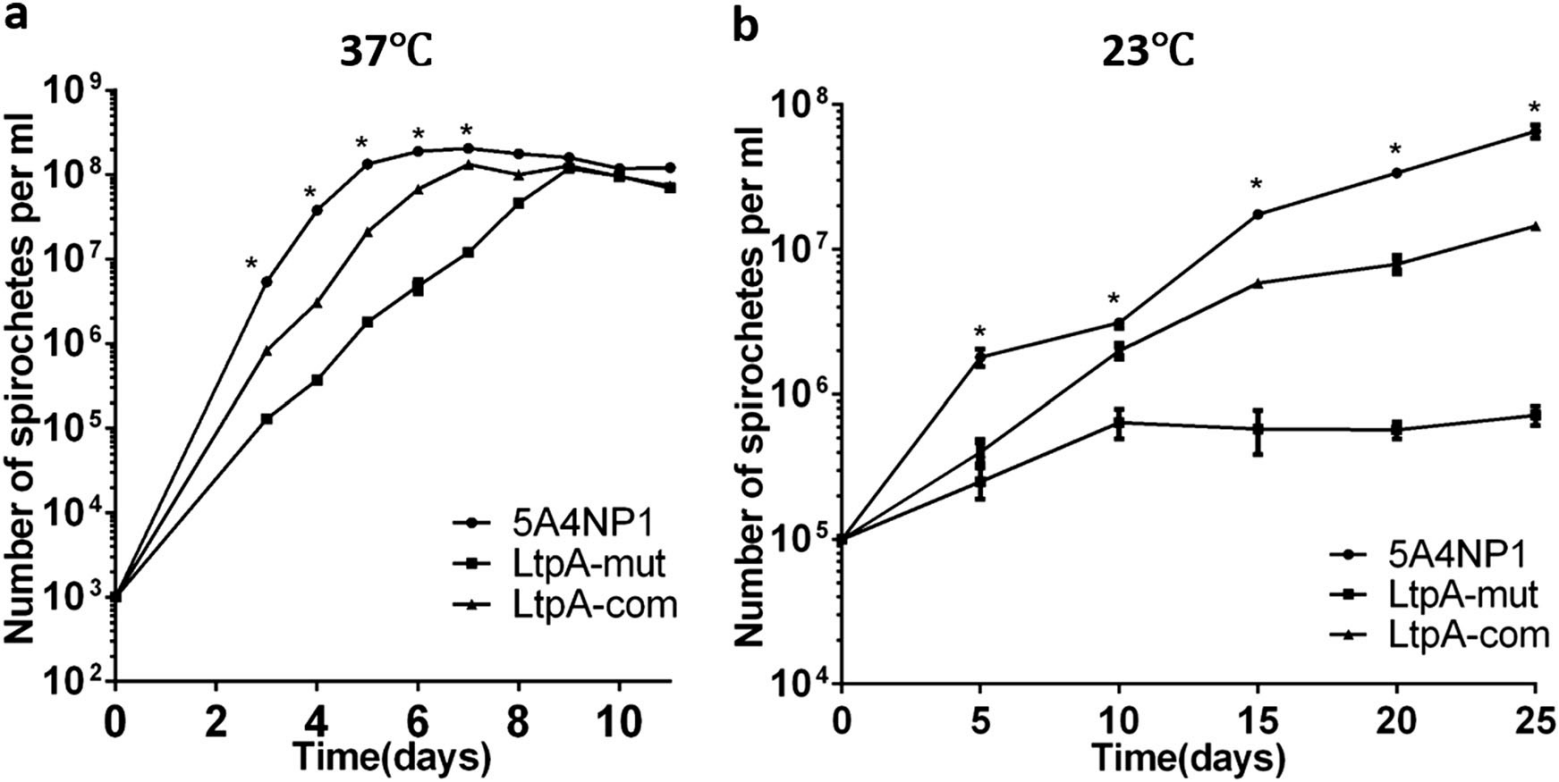
Growth defects in the *ltpA* mutant. WT, LtpA-mut, and LtpA-com strains were cultured in standard BSK-II medium at 37 °C (**a**) or 23 °C (**b**). The initial cell densities for the cultures at 37 °C and 23 °C were 10^3^ spirochetes/ml and 10^5^ spirochetes/ml, respectively. Numbers of spirochetes were enumerated daily using dark-field microscopy. Each data point is derived from the average of the data from three independent cultures. Statistical significance was calculated between LtpA-mut and the WT group. *P < 0.05

The success in generating a *ltpA* mutant suggests that, unlike other CdnL proteins, LtpA is not essential for cell viability. To further examine whether the *ltpA* mutant has defects in vitro, wild-type spirochetes (WT), the *ltpA* mutant, and the complementation strain were cultured at 37 or 23 ℃, and their growth rates were compared. As shown in Fig. 3a, the *ltpA* mutant exhibited slower growth at 37 ℃ than did the wild-type or complemented strain. At 23 ℃, the *ltpA* mutant demonstrated a cold-sensitive phenotype, as it was viable but could hardly replicate under such conditions (Fig. 3b). These observations indicate that LtpA is required for the optimal growth of *B. burgdorferi* in vitro and that its function is more important at lower temperatures mimicking those typical of tick infection.

### The *ltpA* mutant has reduced expression of 16S rRNA

**Fig. 4:**
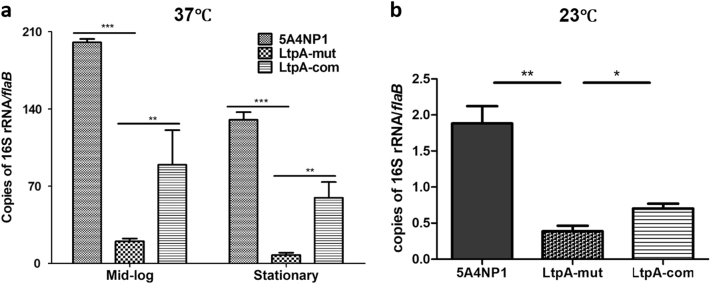
The defect of 16S rRNA expression in the *ltpA* mutant. WT, LtpA-mut, and LtpA-com strains were cultured in standard BSK-II medium at 37 °C (**a**) or 23 °C (**b**). For the culture at 37 °C, spirochetes were collected at the mid-log (10^7^ spirochetes/ml) or stationary phases (5 × 10^8^ spirochetes/ml) and subjected to qRT-PCR analysis. For the culture at 23 °C, spirochetes were collected at a cell density of 10^7^ spirochetes/ml. Each data point was the average of the data from three independent cultures. *, p < 0.05; **,  p < 0.01; ***, p < 0.001

It was reported that CdnL is involved in regulating ribosomal RNA transcription^5^. To investigate whether LtpA influences rRNA transcription in *B. burgdorferi*, which may account for the growth defects and the decreased growth rate, we compared the 16S rRNA levels among the wild-type, *ltpA* mutant, and complemented strains. The results showed that the deletion of *ltpA* markedly reduced the 16S rRNA levels in spirochetes grown at 23 or 37 °C (Fig. 4). The complementation of the *ltpA* mutant with an IPTG-inducible promoter-driven *ltpA* partially restored the 16S rRNA level (Fig. 4).

### The *ltpA* mutant exhibits delayed infection in mice

To examine the *ltpA* mutant’s ability to infect mice, groups of C3H/HeN mice were needle inoculated with the wild-type, *ltpA* mutant, or complemented strain with a dose of 1 × 10^5^ spirochetes per mouse. Mice inoculated with the complementation strain were given IPTG via intraperitoneal (IP) injection in every two days. Ear-punch biopsies were collected and then cultured to determine the presence of spirochetes at different time points. As shown in Table 1, the mice inoculated with wild-type *B. burgdorferi* were culture-positive 2 weeks after inoculation, but no mice were infected when inoculated with the *ltpA* mutant. At 4 weeks after infection, some of the mice infected with the *ltpA* mutant became culture-positive. At week 7, all the mice were killed, and several mouse tissues including skin, bladder, heart, and joint were collected and cultured; seven out of the nine mice inoculated with the *ltpA* mutant were culture-positive (Table 1). These data demonstrated that the *ltpA* mutant is capable of establishing infection in mice, but it is delayed in doing so.

**Table 1:** Mouse infection of the LtpA-defective mutant

C3H/HeN	No. of infected mice/Total no. of mice			
	Needle infection^a^			Tick bite^a^
	2 W	4 W	7 W	7 W
WT	5/5	5/5	5/5	3/3
LtpA-mut	0/9	3/9	7/9	0/6
LtpA-com	1/3	3/3	3/3	1/2

^a^The dose used for needle infection was 10^5^ spirochetes/mouse. For tick bites, 12 infected nymphal ticks were used for each mouse. Time points for ear-punch biopsies or tissue collections were at 2 weeks (2 W), 4 weeks (4 W), or 7 weeks (7 W) after inoculation

### LtpA is required for optimal survival in ticks

**Fig. 5:**
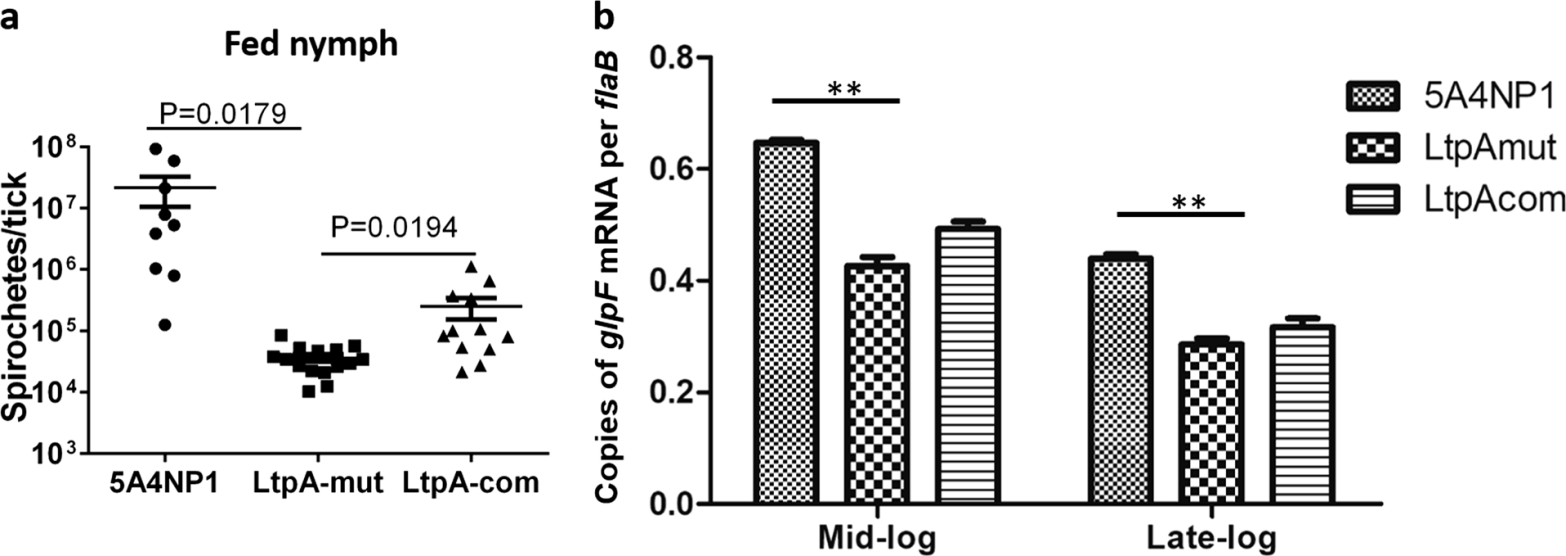
Decreased spirochetal load and *glpF* expression in the *ltpA* mutant. **a** qPCR analysis of spirochetal burdens in fed nymphs. Flat nymphal ticks were first infected with equal amounts of wild-type, *ltpA* mutant, and complementation strains of *B. burgdorferi* by microinjection directly into the midgut of nymphal ticks. Ticks were then allowed to feed on naive mice, and fed nymphal ticks were collected and examined using qPCR analysis. The number of copies of the *B. burgdorferi flaB* gene was chosen to represent spirochete numbers. Each data point represents *flaB* copies in one nymph tick. **b** Transcript levels of *glpF* in WT, LtpA-mut, and LtpA-com strains as determined using qRT-PCR. RNAs were isolated from mid-logarithmic phase and stationary phase cultures grown at 37 °C in a modified BSK-glycerol medium. The *glpF* levels were normalized to *flaB* levels. Values represent the average copy number from three independent cultures. **P < 0.01

To investigate the role of LtpA in *B. burgdorferi* in ticks, equal amounts of the wild-type, *ltpA* mutant, or complemented strains of *B. burgdorferi* were microinjected into the midgut of nymphal ticks. The medium used for the complementation strain contained 1 mM IPTG to ensure the expression of *ltpA* in ticks. No differences in spirochetal numbers, as assessed using qPCR, in ticks prior to feeding was observed (data not shown). Microinjected unfed ticks were fed on naive C3H/HeN mice until fully engorged. The mice used for feeding ticks harboring the complementation strain were given IPTG daily by IP injection during the process of tick feeding. The engorged ticks were collected, and qPCR was used to assess their spirochetal loads. The results showed that the *ltpA* mutant produced a thousandfold lower number of spirochetes in ticks than did wild-type *B. burgdorferi* (Fig. 5a). The spirochetal load was partially restored in the complementation strain. These data indicate that LtpA is required for the optimal survival of ticks upon feeding.

### The *ltpA* mutant is unable to infect mice via tick bites

To examine the *ltpA* mutant’s ability to infect mice during tick infestation, the mice that were used to feed ticks during the above experiment (Fig. 4a) were monitored for infection. The mice infected with ticks carrying the complemented strain were given IPTG by IP injection every two days. All of the ear-punch biopsy cultures from the mice infected with ticks carrying the wild-type *B. burgdorferi* were positive, but all the cultures from the mice infected with ticks carrying the *ltpA* mutant were negative (Table 1). This finding remained true even at 7 weeks post-tick feeding (Table 1). These data suggest that, although the *ltpA* mutant was able to survive within the tick midgut, it was not able to infect mice via tick bites.

The glycerol uptake and metabolic pathway (encoded by the *glpFKD* operon) is known to be important for optimal spirochetal growth in ticks. Thus, we investigated whether LtpA has a role in *glpFKG* expression. The results showed that the *ltpA* mutant exhibited a moderate, although statistically significant, reduction in *glpF* expression in spirochetes compared to that in the wild-type *B. burgdorferi* (Fig. 5b).

## Discussion

*B. burgdorferi* encounters drastic environmental changes when it migrates between the tick vector and the mammalian host. While major advances have been made in understanding how *B. burgdorferi* modulates its gene expression during mammalian host adaptation, few of the regulators required for spirochetal adaptation and survival in ticks have been identified. In this study, we focused on the CarD-like regulator in *B. burgdorferi*, LtpA. CarD is a new family of global transcriptional regulators. Our knowledge of the CarD family remains sparse and is largely gleaned from studies on *Mycobacteria* and *Myxococcus*. We demonstrated that LtpA has an important role in the optimal fitness of *B. burgdorferi* in ticks and is indispensable for the successful transmission of *B. burgdorferi* from ticks to mammals.

The inactivation of *ltpA* in virulent strains of *B. burgdorferi* proved challenging. Multiple attempts to generate an *ltpA* mutant were not successful^30^, and the published transposon library of *B. burgdorferi* also did not yield a transposon insertion within the *ltpA* gene^31^. Two factors likely contributed to our present success in constructing the *ltpA* mutant in this study. First, we used the limiting dilution method instead of using the semisolid agar plating method when selecting the mutant clones after transformation^32–34^. Second, we used the *B. burgdorferi* strain B31 clone 5A4NP1^35^, rather than the strain 297 AH130 that was used previously^30^.

CdnL-type CarD of *M. tuberculosis* and CdnL of *M. Xanthus* are the most studied members of the CdnL family and have been demonstrated to be essential for cell viability^2,5^. For *B. cereus*, inactivation of *cdnL* did not affect cell replication; however, this bacterium has two copies of CdnL^11^. Thus, whether CdnL is required for cell viability in *B. cereus* remains to be determined, which requires inactivation of both copies of *cdnL* genes. In this regard, LtpA is unique, as it is dispensable for spirochetal viability in vitro. On the other hand, the *ltpA* mutant of *B. burgdorferi* showed growth defects, and such defects were markedly augmented at lower temperature, resulting in a cold-temperature-sensitive phenotype. Interestingly, a recent report showed that CdnL was also not essential for cell viability in *Caulobacter crescentus*, and the *cdnL* mutant used in the study was also cold-sensitive^36^.

The growth defects present in the *ltpA* mutant were likely due to decreased rRNA transcription (Fig. 4). Supporting this conclusion, the CdnL-type CarD of *M. tuberculosis* was reported to regulate rRNA expression^5,37,38^. CarD in *M. tuberculosis* is broadly distributed on a number of promoters and uses a minor groove wedge mechanism to stabilize the RNAP transcription complex during transcription initiation^37^. CdnL of *C. crescentus* was also shown to interact with the RNAP β subunit and to localize to at least one rRNA promoter in vivo^36^. A highly conserved tryptophan residue was thought to wedge into the minor groove in the upstream DNA sequence to stabilize the RNAP complex^9^. In addition, CdnL was shown to be capable of binding to DNA, despite lacking the AT-hook DNA-binding domain; a protein crystal structure revealed that the unique C-terminal domain of CdnL consists of a five α-helical fold, which has been shown to have DNA-binding activity in *M. tuberculosis* and *T. thermilus*^4,7^. CdnL in *C. crescentus* was also shown to interact with DNA, but not in *M. xanthus*^6,36^. LtpA lacks the conserved tryptophan residue. Whether LtpA functions via stabilizing RNAP or LtpA directly interacts with DNA to modulate gene expression remains to be determined.

Low levels of rRNA in the *ltpA* mutant also likely contribute to the spirochetal bacterial burden in ticks and possibly delayed infection in mice. During mouse infection, in addition to reduced growth, the *ltpA* mutant may have reduced infectivity. In this study, only a single dose (10^5^ spirochetes/mouse) was given during the needle infection experiment, which precludes the calculation of the ID_50_ value. Nevertheless, the presence of delayed infection suggests that a higher dose of the *ltpA* mutant is required for infection than for the wild-type *B. burgdorferi* (which requires <100 spirochetes). In ticks, *B. burgdorferi* undergoes maximal growth to reach a high number within the tick gut during blood meal feeding, as only a small number of spirochetes are capable of penetrating through the midgut membrane into the tick hemocoel^13,39^. Thus, optimal growth in ticks is important for *B. burgdorferi* to complete the enzootic cycle. Although the *ltpA* mutant was capable of surviving in ticks, it had reduced spirochetal loads after tick feeding. One caveat of this study is that we did not examine the long-term survival in unfed ticks. A reduced spirochetal number in ticks is likely to be one major factors contributing to the inability to infect mice via tick infection. The reduced spirochetal number in ticks with the *ltpA* mutant is the result of low 16S rRNA levels; the defect in *glpF* expression in the *ltpA* mutant is another contributor. In this study, we only examined glpF expression at 37 °C, as the *ltpA* mutant failed to grow at 23 °C (Fig. 3b). Since *glpF* expression is increased by glycerol at both 23 and 37 °C (i.e., regulated similarly by glycerol in both temperature conditions), it is reasonable to postulate that LtpA also positively affected *glpF* expression at a lower temperature, as observed at 37 °C. In addition to reduced growth fitness in ticks, the *ltpA* mutant may also be defective in the process of transmission from tick midgut to the hemocoel, the salivary grand, and eventually to mice. Further understanding of how LtpA regulates gene expression and modulates spirochetal adaptation in ticks will improve our understanding of the molecular mechanisms underlying the enzootic cycle of *B. burgdorferi*.

## Materials and methods

### *B. burgdorferi* strains and culture conditions

*B. burgdorferi* strains (Table 2) were constructed using the low-passage clone LP B31 (strain 5A4NP1), kindly provided by Dr. Steve Norris. Spirochetes were cultivated in complete Barbour–Stoenner–Kelley-II (BSK-II) medium^40^ at 37 or 23 °C with 5% CO_2_.

**Table 2:** Strains, plasmids, and primers used in this study

Strains, plasmids, or primers	Description or sequence	Purpose or source
Strains		
5A4NP1	Wild-type strain with *bbe02* gene disrupted by Kan^r^	35
LtpA-mut	*ltpA* deletion mutant; 5A4NP1 transformed with pXY301R	This study
LtpA-com	*ltpA* complementation; LtpA-mut transformed with pXW006	This study
Plasmids		
pXY301R	Suicide vector for producing *ltpA* deletion; Str^r^	This study
pXW006	Shuttle vector carrying IPTG-inducible *ltpA* gene; Gen^r^	This study
Primers		
qPCR-flaB-F	CAGCAATAGCTTCATCTTGGTTTG	qPCR of *flaB*
qPCR-flaB-R	ACCAGCATCTTCAGGGTCTCA	qPCR of *flaB*
Del-CarD-UF	GCGGCCGCACCATCTTGTTAATGTAAGACG	Amplification of *ltpA* upstream region
Del-CarD-UR	AGATCTCCTTAATCGTACCTACTCCATGC	Same as above
Del-CarD-DF	ACTAGTGTTAGCAGGGAAAAGGTAGAAGA	Amplification of *ltpA* downstream region
Del-CarD-DR	GGTACCTGAAACAAAACCAAATAAATAGG	Same as above
NdeI-priFX-CarDf	GCCGCATATGATGTTGTTCTCAGGAAAA	Amplification of *ltpA* with *Nde*I and *Asc*I sites
AscI-priFX-CarDr	ATTAGGCGCTTACTCACTTTCCCCTAA	Same as above

### Sodium dodecyl sulfate-polyacrylamide gel electrophoresis (SDS-PAGE) and immunoblotting

Spirochetes were harvested by centrifugation at 8000×*g* for 10 min and washed three times with PBS (pH 7.4) at 4 °C. Pellets were suspended in SDS buffer containing 50 mM Tris-HCl (pH 8), 0.3% sodium dodecyl sulfate (SDS), and 10 mM dithiothreitol (DTT). Cell lysates (5 × 10^7^ cells per lane) were separated by 12% SDS-polyacrylamide gel electrophoresis (PAGE) and transferred to nitrocellulose membranes (GE-Healthcare, Milwaukee, WI). Membranes were blotted with antibodies against FlaB (monoclonal, 1:1000 dilution), LtpA (polyclonal, 1:2000 dilution)^30,41^, and then incubated with goat anti-mouse lgG-HRP secondary antibody (1:1000, Santa Cruz Biotechnology, Santa Cruz, CA) or goat anti-rat lgG-HRP secondary antibody (1:2000, Santa Cruz Biotechnology, Santa Cruz, CA). The detection of horse radish peroxidase activity was determined using the enhanced chemiluminescence method (Thermo Pierce ECL Western Blotting Substrate, Waltham, MA) and subsequently by exposure to X-ray film.

### Generation of the *ltpA (bb0355)* mutant and complementation strain

To construct a suicide vector for the inactivation of *ltpA*, regions of DNA corresponding to 1.5 kb upstream and 1.3 kb downstream of *ltpA* were PCR amplified from 5A4NP1 genomic DNA using the primer pair Del-CarD-UF and Del-CarD-UR, and the pair Del-CarD-DF and Del-CarD-DR, respectively (Table 2). The resulting DNA fragments were then cloned upstream and downstream of an *aadA* streptomycin-resistant marker within the suicide vector pXY301R^16^, resulting in pXY301-carD. To construct a shuttle vector for complementation, we first constructed a plasmid carrying a *flaB* promoter-driven *ltpA*. *ltpA* flanked by *Nde*I and *Asc*I restriction sites was amplified using primers NdeI-priFX-CarDf and AscI-priFX-CarDr and cloned into pBSVG downstream of the *flaB* promoter. The *Nde*I and *Aat*II fragment containing the *flgB* promoter-driven *ltpA* and the gentamycin-resistance cassette was cloned into the pOY112 inducible shuttle vector^42^, resulting in the shuttle vector used for complementation, pXW006.

### Mouse infections

All tick-mouse experiments were approved by the IACUC committee of Indiana University School of Medicine under the protocol number #19792. Four-week-old C3H/HeN mice (Harlan, Indianapolis, IN) were subcutaneously inoculated with 1 × 10^5^ spirochetes. For the mice infected with the complemented strain of the *ltpA* mutant, IPTG was given via IP injection (100 µl of 10 mM IPTG) every two days. The mice were killed, and ear, skin (inoculation site), bladder, heart, and joint tissues were collected 7 weeks after infection and cultivated in 2 ml of BSK-II medium (Sigma-Aldrich, St. Louis, MO) containing an antibiotic mixture of phosphomycin (2 mg/ml), rifampin (5 mg/ml), and amphotericin B (250 mg/ml) (Sigma-Aldrich, St. Louis, MO). All cultures were maintained at 37 °C and examined for the presence of spirochetes by dark-field microscopy beginning 5 days after inoculation. A single growth-positive culture was used as the criterion to determine the presence of mouse infection.

### Microinjection of *B. burgdorferi* into nymphal ticks and mouse infection by *B. burgdorferi*

Microinjection and the tick-mouse experiments were approved by the IACUC committee of Indiana University School of Medicine under the protocol number #19792. *I. scapularis* nymphs were obtained from the Tick Rearing Facility at Oklahoma State University (Stillwater, OK). Microinjection was used to introduce spirochetes into the gut of *I. scapularis* nymphs as previously described^34^. Briefly, each *B. burgdorferi* variant was cultivated under normal conditions in BSK-II medium in the presence of corresponding selective antibiotics. Spirochetes were harvested by centrifugation and concentrated in BSK-II medium to a density of 3 × 10^8^ spirochetes/mL. A total of 10 μL of the cell suspension was then loaded into a 1-mm diameter glass capillary needle (World Precision Instruments Inc., Sarasota, FL) using a microloader (Eppendorf AG, Hamburg, Germany). The bacterial suspension was then injected into the rectal aperture of unfed nymphal ticks using a FemtoJet microinjector system (Eppendorf AG, Hamburg, Germany). The parameters for injection were a pressure of 1000 hPa, injection time of 0.1 s, and a compensation pressure of 0 hPa, which delivered an average volume of 0.1 μl (~10^4^ spirochetes). After microinjection, the ticks were placed on adult C3H/HeN mice (12 ticks per mouse), allowed to feed to repletion (4–5 days), and then collected for DNA extraction. Mouse infection was determined using the same procedure used during needle infection.

### Extract DNA from ticks

DNA was isolated from engorged nymphs using the DNeasy® Blood & Tissue Kit B (QIAGEN, Valencia, CA) according to the manufacturer’s instructions. Spirochete burdens within infected ticks were assessed by qPCR using primer pair qflaB-F/R and qTactin-F/R (Table SI). Absolute copy numbers of *flab* are quantified as spirochete loads in ticks.

### Quantitative RT-PCR (qRT-PCR) and qPCR

RNA samples were extracted from *B. burgdorferi* cultures using the RNeasy mini kit (Qiagen, Valencia, CA) according to the manufacturer’s protocol. Three independent culture samples were used for each strain. The digestion of contaminating genomic DNA in RNA samples was performed using RNase-free DNase I (Promega, Madison, WI), and the removal of DNA was confirmed by PCR amplification of the *B. burgdorferi flaB* gene. cDNA was synthesized using SuperScript III reverse transcriptase with random primers (Invitrogen, Carlsbad, CA). To quantify the transcript levels of genes of interest, an absolute quantitation method was used to create a standard curve for the qPCR assay according to the manufacturer’s protocol (Strategene, La Jolla, CA). Briefly, the PCR product of the *flaB* gene served as a standard template. A series of tenfold dilutions (10^2^–10^7^copies/ml) of the standard template was prepared, and qPCR was performed to generate a standard curve by plotting the initial template quantity against the Ct values for the standards. The quantity of the targeted genes in the cDNA samples was calculated using their Ct values and the standard curve. The samples were assayed in triplicate using the ABI 7000 Sequence Detection System and GREEN PCR Master Mix (ABI, Pleasanton, CA). The levels of the target gene transcript were reported as per 100 copies of *flaB*.

### Data availability

The datasets generated and/or analyzed during the current study are available from the corresponding author upon request.
